# Physical activity and depression of Chinese students in Korea: self-efficacy as a mediator and social support as a moderator

**DOI:** 10.3389/fpsyg.2025.1662687

**Published:** 2025-09-22

**Authors:** Pengfei Chang

**Affiliations:** Department of Exercise Prescription, Dongshin University, Naju, Republic of Korea

**Keywords:** depression, international students, physical activity, self-efficacy, social support

## Abstract

**Introduction:**

This study, grounded in social cognitive theory, investigates the impact of physical activity on depression levels among international students, with a focus on the mediating role of self-efficacy and the moderating effect of social support, aiming to unpack the mechanisms linking physical activity to mental health in this cross-cultural population.

**Methods:**

A cross-sectional design was employed, involving 903 international students from two South Korean universities; data were collected via standardized questionnaires and analyzed using SPSS 26.0, with analytical methods including descriptive statistics, correlation analysis, mediation analysis, and moderated regression.

**Results:**

Three key findings were revealed: (1) physical activity exhibited a significant negative correlation with depression levels; (2) physical activity was positively associated with self-efficacy (which in turn negatively correlated with depression), and self-efficacy partially mediated the relationship between physical activity and depression; (3) social support moderated the effect of physical activity on depression.

**Discussion:**

These findings elucidate the underlying mechanisms through which physical activity benefits mental health, highlighting the synergistic roles of self-efficacy (as an internal psychological resource) and social support (as an external buffering factor); the study provides empirical support for integrated interventions combining physical activity promotion, self-efficacy enhancement, and social support reinforcement to mitigate depression among international students, and demonstrates that enhancing self-efficacy and reinforcing social support can significantly amplify the antidepressant effects of physical activity in this group, offering new insights for cross-cultural mental health interventions.

## 1 Introduction

Against the backdrop of China’s evolving economic landscape and increasingly competitive job market, a growing number of university students are pursuing overseas education to access superior academic resources and enhance career prospects. China remains one of the largest sources of international students globally, with a notable trend: an increasing proportion of Chinese students are opting for Asian countries—particularly South Korea—over traditional English-speaking destinations (Lee, 2017). South Korea has emerged as a preferred choice for students from middle-income Chinese families, owing to its academic reputation, cost-effectiveness, and geographical proximity. However, the cross-cultural adaptation process presents multifaceted challenges, including language barriers, cultural differences, and academic pressures, which often lead to significant psychological distress. Research indicates that acculturative stress correlates strongly with depression, psychological resilience, and social support, with depression being the strongest predictor of adaptation difficulties among Chinese students (Kim et al., 2010). Key stressors include culture shock, homesickness, inadequate social support systems, limited cross-cultural social competence, and potential exposure to stereotypes or prejudice. These cumulative pressures may trigger depressive symptoms and other adverse psychological outcomes, ultimately compromising academic performance and the overall cross-cultural educational experience.

Depression is a prevalent mental health disorder characterized by persistent sadness, anhedonia (loss of interest or pleasure), feelings of worthlessness or excessive guilt, sleep or appetite disturbances, fatigue, impaired concentration, and recurrent suicidal ideation. It can affect individuals of any age, gender, race, or socioeconomic status (Kessler et al., 2003). As one of the most common mental illnesses, its prevalence serves as a key indicator of population mental health. Depression represents a global public health concern, with a lifetime prevalence of 16.2% and an annual prevalence of 6.6% in the general population (Ebert et al., 2019). Major depressive disorder (MDD) is particularly prevalent among university students and ranks among the leading causes of disability worldwide (Steptoe et al., 2007). The university years mark a critical transitional period from adolescence to adulthood, during which students face significant life decisions while coping with substantial stressors, including financial pressures, academic demands, and interpersonal challenges (Liu et al., 2019). A longitudinal study tracking 1,401 Chinese undergraduates over four years revealed that 20–40% exhibited varying degrees of depression, anxiety, or stress, with approximately 35% reporting depressive symptoms above normative levels (Wilburn and Smith, 2005). Notably, depression is a well-established risk factor for suicide among adolescents and young adults (De Moor et al., 2006). For international students, the challenges of cross-cultural adaptation further exacerbate psychological distress. Acculturative stress arises as they navigate new environments and reconcile cultural conflicts. Substantial evidence indicates a significant positive correlation between acculturative stress and depression (Hovey, 2000; Tummala-Narra et al., 2012; Revollo et al., 2011; Park and Rubin, 2012). Given these findings, systematic research on depression among international students holds both theoretical and practical significance. Such studies can elucidate the mental health mechanisms underlying cross-cultural adaptation while providing empirical support for targeted interventions.

Physical activity is defined as any bodily movement generated by skeletal muscle contractions that requires energy expenditure. Beyond its well-established benefits for physical health, accumulating evidence demonstrates its significant positive impact on psychological well-being (Mahindru et al., 2023). As early as, Mammen and Faulkner (2013) conducted a systematic review of prospective observational studies to examine the impact of physical activity on depression incidence. Their findings indicated that exercise at any intensity may help prevent subsequent depression (Mammen and Faulkner, 2013). Beyond its critical role in maintaining physical and mental health, physical inactivity poses adverse economic consequences at a national level (Dhuli et al., 2022). Existing evidence suggests a significant correlation between physical activity and depressive symptoms, underscoring the importance of regular exercise. However, despite extensive research on the efficacy of physical activity in mitigating depression, the underlying mechanisms of its antidepressant effects remain unclear. Current investigations aim to elucidate the psychosocial and biological pathways through which physical activity influences mental health in young populations (Lubans et al., 2016).

Self-efficacy, a central construct in Bandura (2013) social cognitive theory, refers to an individual’s perceived capability to execute desired courses of action. It represents one’s confidence in successfully performing necessary actions to meet situational demands. Empirical evidence demonstrates that these efficacy judgments serve as crucial determinants influencing activity selection, effort expenditure, and persistence when confronting failure or adverse stimuli (Bandura, 2013). The self-efficacy theory adopts a social-cognitive perspective to explain behavioral causation, proposing that behavioral, physiological, and cognitive factors interact reciprocally with environmental influences (Bandura, 2013). A strong sense of self-efficacy can help us face challenges and persevere in pursuing our goals, but low self-efficacy can have the opposite effect, leading to avoidance behavior and negative emotions, which not only harm our performance but also damage our physical and mental health(Waddington, 2023).

Social support, derived from interpersonal networks including family, friends, and community, serves as an external protective factor (Awang et al., 2014). Substantial evidence indicates that social support not only promotes psychological well-being but also buffers against stressful life events (Dollete et al., 2004). Students reporting lower-quality social support (as measured by the Multidimensional Scale of Perceived Social Support) demonstrate significantly poorer mental health outcomes. Notably, these individuals demonstrate a sixfold increased risk of developing depressive symptoms relative to peers with robust social support networks (Hefner and Eisenberg, 2009). Given the robust effect size of the correlation between social support and mental health, enhancing social support should be prioritized—particularly for vulnerable populations such as women, older adults, patients, workers, and students (Harandi et al., 2017).

This study aims to address three key questions:

1. Does physical activity significantly reduce depressive symptoms among international students?
2. Does self-efficacy mediate the association between physical activity and depression levels?
3. Does social support moderate the mediating effect of self-efficacy on depression levels in international students?

To address the research objectives, this paper is organized as follows:

Section 2 presents the theoretical framework and develops the research hypotheses. Section 3 details the methodology, including data collection procedures and analytical approaches. Section 4 reports the study findings. Section 5 discusses the theoretical and practical implications, identifies study limitations and proposes future research avenues.

In a multinational cross-sectional study of 17,348 university students (aged 17–30 years) spanning 23 countries/regions, Steptoe et al. (2007) reported a significantly higher prevalence of severe depressive symptoms among East Asian populations (e.g., Japan, South Korea), with rates reaching 38%—markedly elevated compared to other geographic subgroups. Regular physical activity is critical for both physical and mental health. For instance, prior studies using daily step counts as a measure of physical activity reported inverse correlations between step counts and obesity, diabetes, and depression (Luan et al., 2019; Rossen et al., 2015; Ludwig et al., 2018). A 2011 systematic review demonstrated that exercise and physical activity yield beneficial effects on depressive symptoms comparable to antidepressant treatments (Dinas et al., 2011). Further supporting this, a 2020 prospective study revealed that moderate-to-vigorous physical activity performed at least once weekly was inversely associated with depression scores, regardless of gender. Notably, physical activity negatively correlated with depressive symptoms, and prospective analyses indicated that it predicted reduced depression scores 4 years later (Marques et al., 2020).

Based on these findings, we propose the following hypothesis:

*Hypothesis 1*: Physical activity is negatively associated with depression.

Self-efficacy and anticipated outcomes have been established as critical psychosocial determinants of physical activity adherence (Desharnais et al., 1986). Schwartz and Fish found that skill-training interventions aimed at enhancing self-efficacy may be among the most effective approaches to alleviating depressive symptoms (Schwartz and Fish, 1989). Moreover, empirical evidence from a 2010 study confirmed the mediating roles of self-efficacy and mental health in the association between physical activity and quality of life among ethnically diverse older adults (Paxton et al., 2010). Further supporting this, Pu et al. recruited 535 undergraduates from two universities and administered self-efficacy questionnaires, the Revised Life Orientation Test, and the Self-Rating Depression Scale. Their findings indicated that fostering self-efficacy may help reduce depressive symptoms, suggesting implications for university counseling services (Pu et al., 2017). This aligns with earlier research; a 1989 cross-sectional study reported an inverse correlation between self-efficacy and depression (Comunian, 1989). Collectively, these studies suggest a significant relationship among physical activity, self-efficacy, and depression levels.

Thus, we propose the following hypotheses:

*Hypothesis 2*: Physical activity is positively associated with self-efficacy.

*Hypothesis 3*: Self-efficacy mediates the physical activity–depression relationship.

A study of 115 college students found that those with greater social support exhibited lower stress levels and better adaptation to university life (Friedlander et al., 2007). Empirical evidence suggests a significant negative correlation between social support and psychological distress, including depression and stress (Alimoradi et al., 2014; Bukhari and Afzal, 2017; Kugbey et al., 2015). Further research indicates that adolescents’ vigorous physical activity can be indirectly predicted by self-efficacy through behavioral intention, with this mediation effect being moderated by peer support levels—suggesting that peer support may partially compensate for low self-efficacy (Hamilton et al., 2017). Additionally, social support factors such as traffic safety, natural environments, and sports facilities also play a crucial role. Notably, Maleki Pirbazari et al. (2012)demonstrated that self-efficacy and peer support exert significant direct effects on depressive symptoms, while family support has an indirect influence. Among these factors, self-efficacy showed the strongest association with depression, positioning it as a key variable in depression etiology. Early self-efficacy development in students appears heavily dependent on parental and familial support, whereas students with higher self-efficacy tend to engage more actively in social relationships. Moreover, shared experiences among peers make peer support particularly beneficial in mitigating depressive symptoms. Collectively, these findings suggest that interventions aimed at enhancing self-efficacy and strengthening peer and family support may help reduce and prevent depressive symptoms in college students. Based on prior research, we propose the following hypothesis:

*Hypothesis 4*: Social support moderates the mediation effect of self-efficacy.

**Figure 1:**
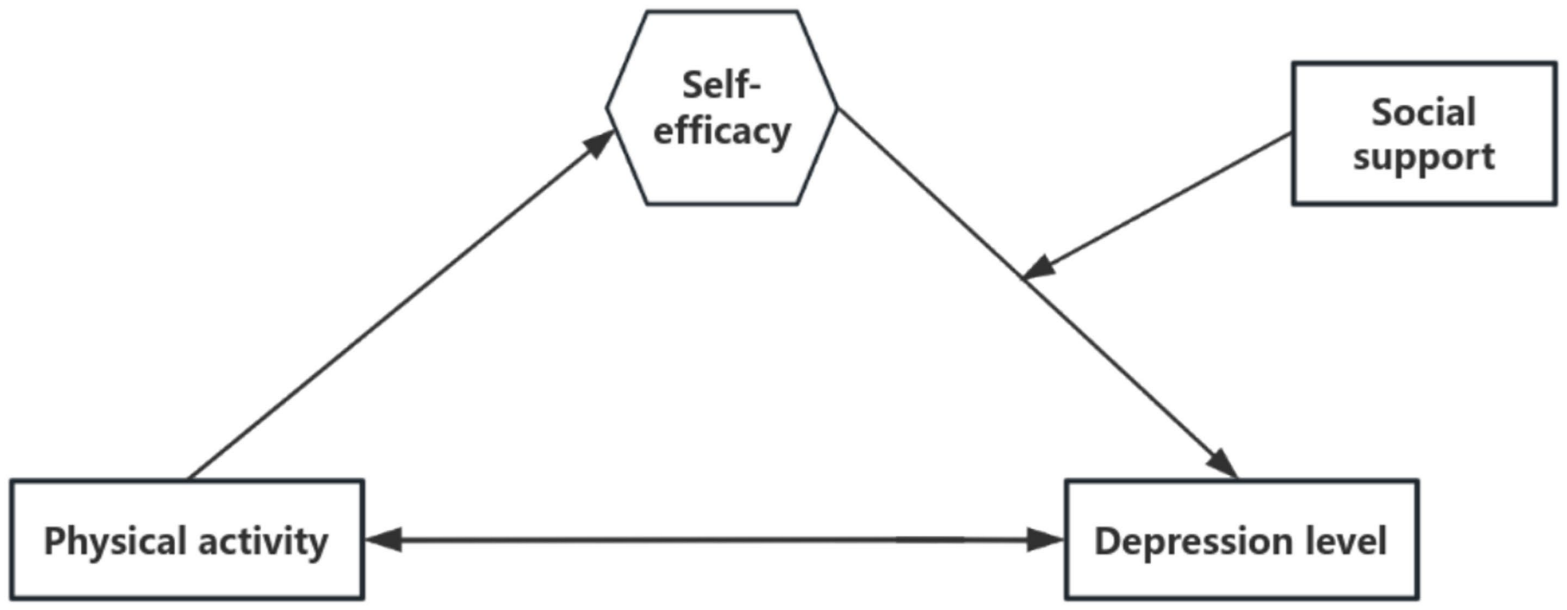
Theoretical framework.

This study investigates the relationship between physical activity and depression among Chinese international students in South Korea. We propose a conceptual framework that examines self-efficacy as a potential mediator in this relationship, while also assessing the moderating role of social support in the associations between physical activity, self-efficacy, and depression (Figure 1).

## 2 Participants and methods

### 2.1 Participants

According to Steptoe et al. (2007), the prevalence of depressive symptoms among Japanese and South Korean university students was 38%. Using a 3% margin of error at the 95% confidence level (Z = 1.96), the minimum required sample size was determined to be 879 participants.

The sample size was calculated using the following formula:


$$n=\frac{Z^{2}\times P\times (1-P)}{e^{2}}$$

Where (n) denotes the sample size, (Z) is the confidence level, (e) is the tolerable error, and (P) is the probability.

Using convenience sampling, we administered questionnaires to Chinese international students at two South Korean universities: Chonnam University (*n* = 368) and Honam University (*n* = 656). Of the 1,024 distributed questionnaires, 903 valid responses were retained after excluding incomplete submissions, yielding a valid response rate of 88.18%. The final analytical sample comprised 903 students with the following characteristics: Gender: female (*n* = 368), male (*n* = 535); Academic level: undergraduate (*n* = 423), master’s (*n* = 357), doctoral (*n* = 123); Only-child status: only children (*n* = 534), non-only children (*n* = 369).

The study employed online questionnaires distributed through Chinese student associations at participating universities, primarily utilizing WeChat groups for dissemination. Prior to participation, respondents received detailed information regarding: (1) the study objectives, (2) data anonymity protocols, and (3) intended data usage. Participants were explicitly informed that the survey involved no foreseeable risks. All participants provided digital informed consent before proceeding. To ensure data quality, we implemented a minimum response time threshold based on questionnaire complexity and length. The electronic survey required approximately 20 min for completion, allowing participants adequate time to provide considered responses.

### 2.2 Methods

In this study, data processing and analysis were performed using SPSS 26. To assess common method bias, Harman’s single-factor test was conducted, and the results indicated no significant bias. The validity of the scales was evaluated using the Kaiser–Meyer–Olkin (KMO) test and Bartlett’s test of sphericity, which confirmed good structural validity. Regression analysis was employed to examine the mediating and moderating effects, and the mediating effect was further verified using the Bootstrap method.

#### 2.2.1 Basic information questionnaire

Demographic data including gender, academic year, only-child status, and urban/rural residency were collected via survey. Given their potential influence on study outcomes, these demographic variables were controlled for in subsequent analyses.

#### 2.2.2 International Physical Activity Questionnaire (IPAQ)

This study employed the International Physical Activity Questionnaire (IPAQ) (Qu and Li, 2004) to assess participants’ physical activity levels. The questionnaire covers six core dimensions: occupational activities, transportation activities, household activities, leisure-time exercise activities, sedentary behavior, and sleep duration. For data processing, we implemented a rigorous quality control protocol: First, the duration of all physical activities was uniformly converted to minutes. Second, records with missing data (duration or frequency) were excluded. A double truncation criterion was applied: For single activities, daily durations exceeding 180 min were recorded as 180 min. Weekly cumulative activity durations exceeding 1,260 min were recorded as 1,260 min. Additionally, to eliminate outliers, data from participants reporting total daily activity durations exceeding 960 min were deemed invalid and excluded. Finally, based on the IPAQ classification standards (Appendix Tables A1 and A2), participants’ physical activity levels were categorized into three groups: high, moderate, and low.

#### 2.2.3 General Self-Efficacy Scale (GSES)

The General Self-Efficacy Scale (GSES), originally developed by German clinical and health psychologist Schwarzer, initially contained 20 items before being refined to 10 items. Currently, the GSES has been translated into more than 20 languages and is widely used internationally (Schwarzer et al., 1999). The Chinese adaptation of the General Self-Efficacy Scale (GSES) was utilized in this investigation.(Zhang and Schwarzer, 1995). The GSES consists of 10 items rated on a 4-point Likert scale: 1 = “Not at all true,” 2 = “Hardly true,” 3 = “Moderately true,” 4 = “Exactly true.”

Higher total scores indicate greater self-efficacy. In this study, the scale demonstrated excellent internal consistency (Cronbach’s α = 0.970).

#### 2.2.4 Perceived Social Support Scale (PSSS)

Zimet et al. (1990) created the original version of the Perceived Social Support Scale (PSSS) (Zimet et al., 1990) and Huang et al. (1996) later modified the scale (Huang et al., 1996), the scale consists of 12 items that gauge respondents’ perceived social support, focusing on three primary areas: family, friends, and significant others. The PSSS utilizes a 7-point Likert scale ranging from 1 (“strongly disagree”) to 7 (“strongly agree”). A representative item states: “When I encounter difficulties, certain individuals (e.g., supervisors, relatives, colleagues) provide genuine comfort and support.” Higher composite scores reflect greater perceived social support. The measure exhibited strong internal reliability in our sample (Cronbach’s *α* = 0.952).

#### 2.2.5 Self-Rating Depression Scale (SDS)

The Self-Rating Depression Scale (SDS), developed by American psychologist Zung (1965), is a self-report instrument designed to assess the severity of depressive symptoms in individuals. The SDS has been widely utilized in clinical screening, research investigations, and mental health assessments, demonstrating established reliability and validity (Zung, 1965). The scale comprises 20 items categorized into four dimensions: affective, somatic, psychological, and behavioral symptoms. Scoring Protocol:10 items are positively scored (1–4 points),10 items are reverse-scored (4–1 points), Standard score = Total raw score × 1.25 (rounded), Chinese normative classification: < 53: No depression, 53–62: Mild, 63–72: Moderate, >72: Severe. The scale exhibited high internal consistency in the current study (Cronbach’s α = 0.83).

## 3 Results

### 3.1 Descriptive analysis

The study included 903 participants in total. The gender distribution comprised 471 males (52.2%) and 432 females (47.8%). Regarding educational attainment, the majority held bachelor’s degrees (*n* = 414, 45.8%), followed by master’s degrees (*n* = 289, 32.0%) and doctoral degrees (*n* = 200, 22.1%). A total of 493 participants (54.6%) were only children. In terms of residential background, 538 participants (59.6%) came from urban areas, while 365 (40.4%) were from rural regions (Tables 1, 2).

**Table 1:** Description of basic information.

Variable	Category	Frequency (*n*)	Percentage (%)
Gender	Male	471	52.2
	Female	432	47.8
Education level	Bachelor’s	414	45.8
	Master’s	289	32
	Doctoral	200	22.1
Only child status	Yes	493	54.6
	No	410	45.4
Residential area	Urban	538	59.6
	Rural	365	40.4

**Table 2:** Information description of basic variables.

Variables	*N* = 903		
	Q25	M	Q75
Occupational activity	1	2	3
Transportation activity	1	2	3
Household activity	1	2	3
Leisure exercise	1	2	3
Sedentary behavior	1	2	3
Sleep duration	1	2	3
Self-efficacy	14	16	31
Social support	23	25	47
Depression	68	70	72
Physical activity	11	13	22

#### 3.1.1 Categorical variable clusters (occupational, transportation, household, and leisure exercise activities, sedentary behavior, sleep duration)

The 25, 50, and 75% percentiles of these six variables consistently followed an increasing sequence of 1, 2, and 3, indicating that they are three-level ordered categorical variables. A median value of 2 suggests that sample participation in these activities was predominantly at a moderate level. The 75th percentile being 3 reflects a distribution constrained within low–medium–high tiers, showing significant central clustering and an absence of extreme values.

#### 3.1.2 Continuous variable distribution characteristics

##### 3.1.2.1 Self-efficacy and social support

Both exhibited a typical right-skewed distribution, characterized by a smaller interquartile range (IQR) between the 25th and 50th percentiles (self-efficacy: 2; social support: 2) compared to that between the 50th and 75th percentiles (self-efficacy: 15; social support: 22). This pattern suggests greater heterogeneity among high-scoring individuals, with a considerable proportion of the sample belonging to high-value groups.

##### 3.1.2.2 Depression

The IQR was uniformly distributed (25th–50th: 2; 50th–75th: 2), indicating low dispersion within the sample. The overall distribution was highly concentrated within the 68–72 range, reflecting minimal individual variability.

##### 3.1.2.3 Physical activity

A mildly right-skewed distribution was observed, with a larger IQR between the 50th and 75th percentiles (9) compared to that between the 25th and 50th percentiles (2). Moderate dispersion was noted in the higher range, suggesting the presence of a subset of individuals with relatively high participation levels.

Analysis revealed that demographic variables such as gender and education level were associated with depression and self-efficacy to varying degrees. For instance, a significant gender difference was observed in physical activity participation, with males demonstrating slightly higher levels of engagement compared to females.

### 3.2 Reliability analysis

Cronbach’s alpha was employed to evaluate the internal consistency reliability of the scale, with values ranging from 0 to 1 (higher values indicating greater reliability). Statistical analysis using SPSS 26 yielded a Cronbach’s alpha coefficient of 0.885, demonstrating good internal consistency and confirming the scale’s appropriateness for subsequent analyses (Table 3).

**Table 3:** Results of confidence analysis.

Reliability statistics	
Cronbach’s α	Number of items
0.885	49

Subsequently, reliability analysis was conducted separately for the items within each of the five dimensions. Overall, all dimensions exhibited relatively high Cronbach’s *α* coefficients (typically around or above 0.8), indicating good internal consistency among the items within their respective dimensions (Table 4).

**Table 4:** Reliability analysis results for each dimension.

Dimension	Item	Number of items	Cronbach’s *α*	Cronbach’s α after deletion
1	Item5	7	0.940	0.93
	Item6			0.933
	Item7			0.931
	Item8			0.931
	Item9			0.93
	Item10			0.931
	Item11			0.931
2	A1	10	0.954	0.950
	A2			0.950
	A3			0.950
	A4			0.949
	A5			0.949
	A6			0.949
	A7			0.949
	A8			0.949
	A9			0.949
	A10			0.950
3	B1	12	0.977	0.975
	B2			0.975
	B3			0.975
	B4			0.975
	B5			0.975
	B6			0.975
	B7			0.975
	B8			0.974
	B9			0.975
	B10			0.975
	B11			0.975
	B12			0.975
4	Item14	20	0.971	0.969
	Item15			0.969
	Item16			0.969
	Item17			0.969
	Item18			0.969
	Item19			0.969
	Item20			0.969
	Item21			0.969
	Item22			0.969
	Item23			0.969
	Item24			0.969
	Item25			0.969
	Item26			0.969
	Item27			0.970
	Item28			0.969
	Item29			0.970
	Item30			0.969
	Item31			0.969
	Item32			0.969
	Item33			0.969

### 3.3 Validity analysis

Using SPSS 26, we conducted the Kaiser-Meyer-Olkin (KMO) test and Bartlett’s test of sphericity to assess the factorability of the scale. The KMO value of 0.982 exceeded the recommended threshold of 0.8, indicating excellent sampling adequacy. Additionally, Bartlett’s test was statistically significant (*p* < 0.01), confirming sufficient inter-item correlations. These results demonstrate that the questionnaire meets validity requirements and is suitable for subsequent analyses (Table 5).

**Table 5:** Validity analysis results.

KMO and Bartlett’s test results		
KMO measure of sampling adequacy		0.982
Bartlett’s test of sphericity	*χ* ^2^	40647.842
	df	1,176
	*p*	0.000

### 3.4 Correlation analysis

Pearson correlation analysis revealed significant positive correlations between physical activity and self-efficacy (*r* = 0.129, *p* < 0.01) as well as social support (*r* = 0.131, *p* < 0.01). A significant negative correlation was observed between physical activity and depression (*r* = −0.548, *p* < 0.01). Furthermore, self-efficacy was positively correlated with social support (*r* = 0.423, *p* < 0.01) and negatively correlated with depression (*r* = −0.204, *p* < 0.01). Social support also showed a significant negative correlation with depression (*r* = −0.266, *p* < 0.01) (Table 6).

**Table 6:** Correlation analysis of variables.

Variables	Sporting activity	Self-efficacy	Social support	Depression
Sporting activity	1			
Self-efficacy	0.129**	1		
Social support	0.131**	0.423**	1	
Depression	−0.548**	−0.204**	−0.266**	1

**p < 0.01 (two-tailed test).

### 3.5 Mediation effect analysis

We performed a mediation analysis with physical activity as the independent variable, depression as the dependent variable, and self-efficacy as the mediator, while controlling for gender, education level, only-child status, and residential area. The results of this analysis are presented in the following Table 7.

**Table 7:** Regression analysis.

Model	Variables	*β*	SE	*t*	*p*
Model 1 (depression)	Gender	0.008	0.794	0.281	0.779
	Education level	0.024	0.502	0.867	0.386
	Only child or not	−0.007	0.796	−0.249	0.804
	Residential area	−0.014	0.808	−0.499	0.618
	Physical activity	−0.549	0.050	−19.631	0.000***
	*R*^2^ = 0.301, *F* = 77.374***				
Model 2 (self-efficacy)	Gender	0.017	0.556	0.507	0.612
	Education level	−0.014	0.352	−0.428	0.669
	Only child or not	0.016	0.557	0.471	0.637
	Residential area	0.026	0.566	0.789	0.430
	Physical activity	0.129	0.035	3.889	0.000***
	*R*^2^ = 0.018, *F* = 3.295**				
Model 3 (depression)	Gender	0.010	0.784	0.366	0.714
	Education level	0.022	0.496	0.809	0.419
	Only child or not	−0.005	0.787	−0.176	0.861
	Residential area	−0.010	0.798	−0.378	0.706
	Physical activity	−0.532	0.050	−19.087	0.000***
	Self-efficacy	−0.135	0.047	−4.840	0.000***
	*R*^2^ = 0.319, *F* = 138.215***				

Significance levels are indicated as follows: *p < 0.1, **p < 0.05, ***p < 0.001.

This table presents the results of three regression models examining the direct effect of physical activity on depression (Model 1), the effect of physical activity on self-efficacy (Model 2), and the combined effects of physical activity and self-efficacy on depression (Model 3).

In Model 1, physical activity was significantly negatively associated with depressive symptoms (*β* = −0.549, *p* < 0.001), indicating that higher levels of physical activity were linked to lower levels of depression. Variables including gender, education level, only-child status, and residential area showed no significant effects on depressive symptoms.

Model 2 demonstrated a significant positive effect of physical activity on self-efficacy (*β* = 0.129, *p* < 0.001), suggesting that greater engagement in physical activity is associated with stronger self-efficacy.

In Model 3, self-efficacy showed a significant negative association with depressive symptoms (*β* = −0.135, *p* < 0.001), supporting its mediating role in the relationship between physical activity and depression. The direct effect of physical activity on depressive symptoms remained significant (*β* = −0.532, *p* < 0.001) (Table 8).

**Table 8:** Analysis of intermediation effects.

Type of effect	Effect	Proportion	95% CI		*p*
			Lower limit	Upper limit	
Direct effect	−0.948	96.83%	−1.045	−0.85	0.000
Intermediary effect	−0.031	3.17%	−0.055	−0.013	0.000
Aggregate effect	−0.979	100%	−1.077	−0.881	0.000

#### 3.5.1 Mediation analysis results

The mediation analysis revealed a significant direct effect of physical activity on depression (*β* = −0.948, accounting for 96.83% of the total effect). The mediation effect through self-efficacy was also significant (*β* = −0.031, representing 3.17% of the total effect), with a 95% bootstrap confidence interval of [−0.055, −0.031] excluding zero. These results confirm the mediating role of self-efficacy in the relationship between physical activity and depression, thereby supporting Hypothesis 3.

### 3.6 Moderation analysis

We examined the moderated mediation effect using Model 14 from Hayes’ PROCESS macro (Version 4.0) while controlling for covariates. To mitigate multicollinearity and enhance interpretability, all primary variables were standardized prior to analysis. The interaction term was computed between the standardized mediator and moderator variables (Table 9).

**Table 9:** Regression analysis.

Model	Variables	*β*	SE	*t*	*p*
Model 1 (self-efficacy)	Gender	0.282	0.556	0.507	0.621
	Education level	−0.150	0.352	−0.428	0.573
	Only child or not	0.263	0.557	0.471	0.836
	Residential area	0.446	0.566	0.789	0.827
	Physical activity	0.136	0.035	3.889	0.000***
	*R*^2^ = 0.018, *F* = 3.295**				
Model 2 (depression)	Gender	0.354	0.776	0.456	0.621
	Education level	0.309	0.491	0.629	0.573
	Only child or not	−0.333	0.782	−0.426	0.836
	Residential area	−0.197	0.790	−0.249	0.827
	Physical activity	−0.930	0.049	−18.868	0.000***
	Self-efficacy	0.051	0.107	0.471	0.001**
	Social support	0.026	0.056	0.456	0.987
	Physical activity*Social support	−0.005	0.002	−2.230	0.036*
	*R*^2^ = 0.335, *F* = 56.375***				

Significance levels are denoted as follows: *p < 0.05, **p < 0.01, ***p < 0.001.

Model 1 examined factors influencing self-efficacy. The results indicated a significant positive effect of physical activity on self-efficacy (*β* = 0.136, *p* < 0.05), suggesting that higher levels of physical activity are associated with stronger self-efficacy.

Model 2 analyzed the effects of various variables on depressive symptoms. Physical activity showed a significant negative association with depressive symptoms (*β* = −0.930, *p* < 0.001), indicating that greater engagement in physical activity is related to lower levels of depression. Furthermore, the interaction term between physical activity and social support also had a significant negative effect on depressive symptoms (*β* = −0.005, *p* < 0.05), suggesting that the protective effect of physical activity against depression is more pronounced under higher levels of social support.

*R*^2^ values indicate the proportion of variance in the dependent variable explained by the model. Model 1 had an *R*^2^ of 0.018, indicating that the included independent variables account for 1.8% of the variance in self-efficacy. Model 2 had an *R*^2^ of 0.335, indicating that 33.5% of the variance in depressive symptoms is explained by the model.

The F-statistic was used to assess the overall significance of each model. Model 1 yielded an *F*-value of 3.295 (*p* < 0.01), and Model 2 yielded an F-value of 56.375 (*p* < 0.001), both indicating that the models are statistically significant overall.

**Figure 2:**
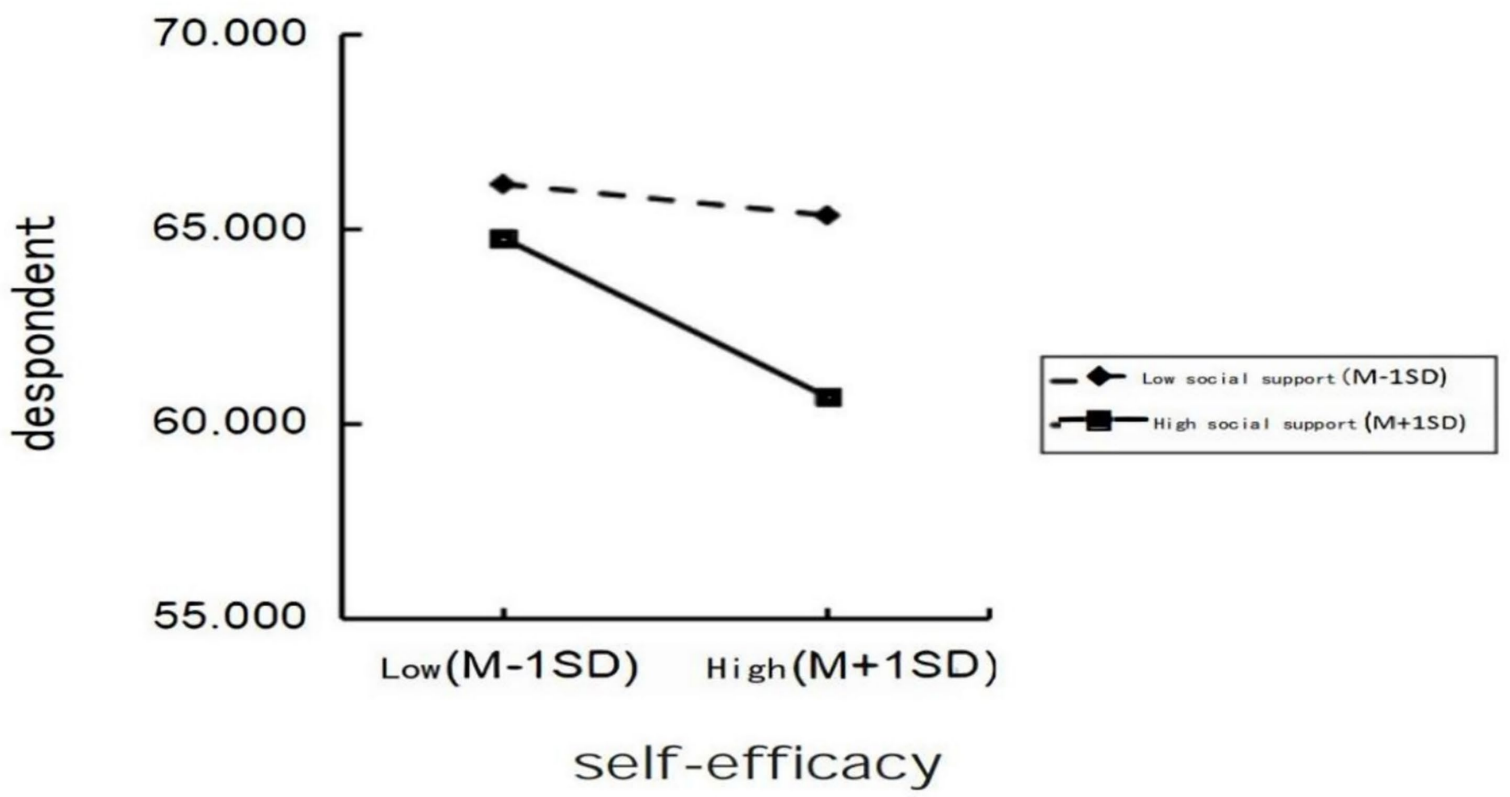
Simple effect diagram.

The simple effects analysis (Figure 2) revealed that as self-efficacy increased, individuals with high social support exhibited lower depression levels compared to those with low social support, indicating that social support serves as a protective factor against depression. These results further support Hypothesis 4.

## 4 Discussion

The present study examined the effects of physical activity on depression levels among international students, examining the mediating role of self-efficacy and the moderating effect of social support. Our results demonstrated a significantly inverse association the relationship linking physical activity and depression, supporting earlier findings. For instance, a 2013 survey of 1,042 adults revealed that sedentary individuals were twice as likely to develop depressive symptoms compared to regular exercisers, with higher prevalence rates of anxiety (9.8%) and depression (10.9%) among inactive participants (De Mello et al., 2013). Of particular importance, self-efficacy was identified as a robust mediating factor in the physical activity-depression association. This aligns with established literature demonstrating positive correlations between self-efficacy and physical activity, and negative associations with depression levels. Previous research involving 1,152 adolescents (Hagger et al., 2001) and subsequent studies by Kavanagh (2014) have consistently identified self-efficacy as a crucial determinant of health behaviors and behavioral modifications. Importantly, the data corroborated our hypothesis that social support moderates the association between self-efficacy and depression. Whereas self-efficacy functions as an internal protective mechanism, social support operates as an external buffering factor. This finding corroborates prior evidence suggesting that supportive environments can influence physical activity levels across individuals with varying self-efficacy (Deforche et al., 2010). Thus, our results substantiate that social support not only enhances the beneficial effects of self-efficacy but also mitigates its negative associations with depression.

### 4.1 Theoretical and practical implications

Despite accumulating evidence supporting the positive effects of physical activity on depression and anxiety (Ströhle, 2009), its clinical application—particularly as an adjunct to established treatments like psychotherapy or pharmacotherapy—remains in its nascent stages. The current study elucidates the mediating role of self-efficacy in the physical activity-depression relationship among international students, while empirically validating the moderating effect of social support. These findings provide theoretical advancements to both exercise psychology and cross-cultural mental health research.

### 4.2 Limitations and recommendations for further study

Although this study focused specifically on Chinese international students to control for cultural variability, this limits the generalizability of the findings to other international student populations. Future research should examine differences in these relationships across different cultural groups to determine whether the observed patterns are universal or culturally specific.

This study has several limitations: first, all participants were international students from the same country and cultural background, which may limit the generalizability of the findings; second, the reliance on self-report questionnaires may introduce subjective memory and interpretation biases; additionally, the study employed a relatively simple model - future research could examine multifactorial mechanisms influencing depression and explore differential effects of exercise types and cultural backgrounds to inform targeted interventions. Based on these limitations, future studies should: 1) conduct cross-cultural comparisons examining psychological adaptation differences among students in various host countries and from different nations; 2) employ multiple assessment methods incorporating objective measures (e.g., activity data from fitness apps); 3) adopt longitudinal tracking or randomized controlled trial designs. These improvements will help establish a more comprehensive international student mental health support system and provide scientific evidence for developing tailored interventions.

## 5 Conclusion

This study confirms that physical activity significantly reduces depression levels among international students. Self-efficacy partially mediates this relationship, while social support moderates the effect of physical activity on depression. These findings highlight the importance of integrated interventions that promote physical activity, enhance self-efficacy, and strengthen social support to improve mental health outcomes in this population.

## Data Availability

The original contributions presented in the study are included in the article/supplementary material, further inquiries can be directed to the corresponding author.

## References

[ref1] Alimoradi M. Asadi H. Asadbeigy H. Asadniya R. (2014). The study of links between social support and psychological problems among youth. Int. J. Basic Sci. Appl. Res. 3, 270–274.

[ref2] Awang M. M. Kutty F. M. Ahmad A. R. (2014). Perceived social support and well being: first-year student experience in university. *International Education Studies*, 7, 261–270.

[ref3] Bandura A. (2013). Self-efficacy: the foundation of agency1[M]//control of human behavior, mental processes, and consciousness. (Eds.) W. J. Freeman, H. Kawanagh, & M. D. Zeiler. Psychology Press (Original work published 2000), 16–30.

[ref4] Bukhari S. R. Afzal F. (2017). Perceived social support predicts psychological problems among university students. Int. J. Indian Psychol. 4, 18–27.

[ref5] Comunian A. L. (1989). Some characteristics of relations among depression, anxiety, and self-efficacy. Percept. Mot. Skills 69, 755–764. doi: 10.1177/00315125890693-109, PMID: 2608390

[ref6] De Mello M. T. de Aquino Lemos V. Antunes H. K. M. Lemos V. d. A. Bittencourt L. Santos-Silva R.. (2013). Relationship between physical activity and depression and anxiety symptoms: a population study. J. Affect. Disord. 149, 241–246. doi: 10.1016/j.jad.2013.01.035, PMID: 23489405

[ref7] De Moor M. H. M. Beem A. L. Stubbe J. H. Boomsma D. I. De Geus E. J. (2006). Regular exercise, anxiety, depression and personality: a population-based study. Prev. Med. 42, 273–279. doi: 10.1016/j.ypmed.2005.12.002, PMID: 16439008

[ref8] Deforche B. Van Dyck D. Verloigne M. De Bourdeaudhuij I. (2010). Perceived social and physical environmental correlates of physical activity in older adolescents and the moderating effect of self-efficacy. Prev. Med. 50, S24–S29. doi: 10.1016/j.ypmed.2009.08.017, PMID: 19818363

[ref9] Desharnais R. Bouillon J. Godin G. (1986). Self-efficacy and outcome expectations as determinants of exercise adherence. Psychol. Rep. 59, 1155–1159. doi: 10.2466/pr0.1986.59.3.1155

[ref10] Dhuli K. Naureen Z. Medori M. C. Fioretti F. Caruso P. Perrone M. A.. (2022). Physical activity for health. J. Prev. Med. Hyg. 63, E150–E159. doi: 10.15167/2421-4248/jpmh2022.63.2S3.2756, PMID: 36479484 PMC9710390

[ref11] Dinas P. C. Koutedakis Y. Flouris A. D. (2011). Effects of exercise and physical activity on depression. Ir. J. Med. Sci. 180, 319–325. doi: 10.1007/s11845-010-0633-9, PMID: 21076975

[ref12] Dollete M. Steese S. Phillips W. Matthews G. (2004). Understanding girls’ circle as an intervention on perceived social support, body image, self-efficacy, locus of control and self-esteem. The journal of psychology, 90(2), 204-215.Experience in university. Int. Educ. Stud. 7, 261–270. 16689441

[ref13] Ebert D. D. Buntrock C. Mortier P. Auerbach R. Weisel K. K. Kessler R. C.. (2019). Prediction of major depressive disorder onset in college students. Depress. Anxiety 36, 294–304. doi: 10.1002/da.22867, PMID: 30521136 PMC6519292

[ref14] Friedlander L. J. Reid G. J. Shupak N. Cribbie R. (2007). Social support, self-esteem, and stress as predictors of adjustment to university among first-year undergraduates. J. Coll. Stud. Dev. 48, 259–274. doi: 10.1353/csd.2007.0024

[ref15] Hagger M. S. Chatzisarantis N. Biddle S. J. H. (2001). The influence of self-efficacy and past behaviour on the physical activity intentions of young people. J. Sports Sci. 19, 711–725. doi: 10.1080/02640410152475847, PMID: 11522147

[ref16] Hamilton K. Warner L. M. Schwarzer R. (2017). The role of self-efficacy and friend support on adolescent vigorous physical activity. Health Educ. Behav. 44, 175–181. doi: 10.1177/1090198116648266, PMID: 27226431

[ref17] Harandi T. F. Taghinasab M. M. Nayeri T. D. (2017). The correlation of social support with mental health: a meta-analysis. Electron. Physician 9, 5212–5222. doi: 10.19082/5212, PMID: 29038699 PMC5633215

[ref18] Hefner J. Eisenberg D. (2009). Social support and mental health among college students. Am. J. Orthopsychiatry 79, 491–499. doi: 10.1037/a0016918, PMID: 20099940

[ref19] Hovey J. D. (2000). Acculturative stress, depression, and suicidal ideation in Mexican immigrants. Cult. Divers. Ethn. Minor. Psychol. 6, 134–151. doi: 10.1037/1099-9809.6.2.134, PMID: 10910528

[ref20] Huang L. Jiang Q. J. Ren W. H. (1996). Study on the correlation between coping style, social support and psychosomatic symptoms of cancer patients. Chin. Ment. Health J. 4, 160–161.

[ref21] Kavanagh D. J. (2014). Self-efficacy and depression[M]//self-efficacy: Taylor & Francis, 177–194.

[ref22] Kessler R. C. Berglund P. Demler O. Jin R. Koretz D. Merikangas K. R.. (2003). The epidemiology of major depressive disorder: results from the National Comorbidity Survey Replication (NCS-R). JAMA 289, 3095–3105. doi: 10.1001/jama.289.23.3095 12813115

[ref23] Kim H. K. Son Y. J. Lee M. R. Lim K. C. Chang H. K. Han S. J.. (2010). Predictors of acculturative stress among Chinese students in Korea. Korean J. Adult Nurs. 22, 143–152.

[ref24] Kugbey N. Osei-Boadi S. Atefoe E. A. (2015). The influence of social support on the levels of depression, anxiety and stress among students in Ghana. J. Educ. Pract. 6, 135–140.

[ref25] Lee S. W. (2017). Circulating east to east: understanding the push–pull factors of Chinese students studying in Korea. J. Stud. Int. Educ. 21, 170–190. doi: 10.1177/1028315317697540

[ref26] Liu X. Ping S. Gao W. (2019). Changes in undergraduate students’ psychological well-being as they experience university life. Int. J. Environ. Res. Public Health 16:2864. doi: 10.3390/ijerph16162864, PMID: 31405114 PMC6719208

[ref27] Luan X. Tian X. Zhang H. Huang R. Li N. Chen P.. (2019). Exercise as a prescription for patients with various diseases. J. Sport Health Sci. 8, 422–441. doi: 10.1016/j.jshs.2019.04.002, PMID: 31534817 PMC6742679

[ref28] Lubans D. Richards J. Hillman C. Faulkner G. Beauchamp M. Nilsson M.. (2016). Physical activity for cognitive and mental health in youth: a systematic review of mechanisms. Pediatrics 138:1642. doi: 10.1542/peds.2016-1642, PMID: 27542849

[ref29] Ludwig V. M. Bayley A. Cook D. G. Stahl D. Treasure J. L. Asthworth M.. (2018). Association between depressive symptoms and objectively measured daily step count in individuals at high risk of cardiovascular disease in South London, UK: a cross-sectional study. BMJ Open 8:e020942. doi: 10.1136/bmjopen-2017-020942, PMID: 29654044 PMC5898324

[ref30] Mahindru A. Patil P. Agrawal V. (2023). Role of physical activity on mental health and well-being: a review. Cureus 15:33475. doi: 10.7759/cureus.33475, PMID: 36756008 PMC9902068

[ref31] Maleki Pirbazari M. Nouri R. Sarami G. (2012). Social support and depression symptoms: the mediating role of self-efficacy. Contemporary Psychology, Biannual Journal of the Iranian Psychological Association 6, 26–34.

[ref32] Mammen G. Faulkner G. (2013). Physical activity and the prevention of depression: a systematic review of prospective studies. Am. J. Prev. Med. 45, 649–657. doi: 10.1016/j.amepre.2013.08.001, PMID: 24139780

[ref33] Marques A. Bordado J. Peralta M. Gouveia E. R. Tesler R. Demetriou Y.. (2020). Cross-sectional and prospective relationship between physical activity and depression symptoms. Sci. Rep. 10:16114. doi: 10.1038/s41598-020-72987-4, PMID: 32999306 PMC7527477

[ref34] Park H. S. Rubin A. (2012). The mediating role of acculturative stress in the relationship between acculturation level and depression among Korean immigrants in the US. Int. J. Intercult. Relat. 36, 611–623. doi: 10.1016/j.ijintrel.2012.04.008

[ref35] Paxton R. J. Motl R. W. Aylward A. Nigg C. R. (2010). Physical activity and quality of life—the complementary influence of self-efficacy for physical activity and mental health difficulties. Int. J. Behav. Med. 17, 255–263. doi: 10.1007/s12529-010-9086-9, PMID: 20449700

[ref36] Pu J. Hou H. Ma R. (2017). Direct and indirect effects of self-efficacy on depression: the mediating role of dispositional optimism. Curr. Psychol. 36, 410–416. doi: 10.1007/s12144-016-9429-z

[ref37] Qu N. N. Li K. J. (2004). Study on the reliability and validity of international physical activity questionnaire (Chinese vision, IPAQ). Zhonghua Liu Xing Bing Xue Za Zhi = Zhonghua Liuxingbingxue Zazhi 25, 265–268. 15200945

[ref38] Revollo H. W. Qureshi A. Collazos F. Valero S. Casas M. (2011). Acculturative stress as a risk factor of depression and anxiety in the Latin American immigrant population. Int. Rev. Psychiatry 23, 84–92. doi: 10.3109/09540261.2010.545988, PMID: 21338303

[ref39] Rossen J. Yngve A. Hagströmer M. Brismar K. Ainsworth B. E. Iskull C.. (2015). Physical activity promotion in the primary care setting in pre-and type 2 diabetes-the Sophia step study, an RCT. BMC Public Health 15, 1–11. doi: 10.1186/s12889-015-1941-9 26164092 PMC4499440

[ref40] Schwartz J. Fish J. M. (1989). Self-efficacy and depressive affect in college students. J. Ration.-Emot. Cogn.-Behav. Ther. 7, 219–236. doi: 10.1007/BF01073809

[ref41] Schwarzer R. Mueller J. Greenglass E. (1999). Assessment of perceived general self-efficacy on the internet: data collection in cyberspace. Anxiety Stress Coping 12, 145–161. doi: 10.1080/10615809908248327

[ref42] Steptoe A. Ardle J. Tsuda A. Wardle J. (2007). Depressive symptoms, socio-economic background, sense of control, and cultural factors in university students from 23 countries. Int. J. Behav. Med. 14, 97–107. doi: 10.1007/BF03004175, PMID: 17926438

[ref43] Ströhle A. (2009). Physical activity, exercise, depression and anxiety disorders. J. Neural Transm. 116, 777–784. doi: 10.1007/s00702-008-0092-x, PMID: 18726137

[ref44] Tummala-Narra P. Alegria M. Chen C. N. (2012). Perceived discrimination, acculturative stress, and depression among south Asians: mixed findings. Asian Am. J. Psychol. 3, 3–16. doi: 10.1037/a0024661

[ref45] Waddington J. (2023). Self-efficacy. ELT J. 77, 237–240. doi: 10.1093/elt/ccac046

[ref46] Wilburn V. R. Smith D. E. (2005). Stress, self-esteem, and suicidal ideation in late adolescents. Adolescence 40, 33–45. 15861616

[ref47] Zhang J. X. Schwarzer R. (1995). Measuring optimistic self-beliefs: a Chinese adaptation of the general self-efficacy scale. Psychologia 38, 174–181.

[ref48] Zimet G. D. Powell S. S. Farley G. K. Werkman S. Berkoff K. A. (1990). Psychometric characteristics of the multidimensional scale of perceived social support. J. Pers. Assess. 55, 610–617. doi: 10.1080/00223891.1990.9674095 2280326

[ref49] Zung W. W. K. (1965). A self-rating depression scale. Arch. Gen. Psychiatry 12, 63–70. 14221692 10.1001/archpsyc.1965.01720310065008

